# The SWI/SNF subunit Bcl7a contributes to motor coordination and Purkinje cell function

**DOI:** 10.1038/s41598-017-17284-3

**Published:** 2017-12-06

**Authors:** Lena Wischhof, Simona Maida, Antonia Piazzesi, Anna Gioran, Kristina Barragan Sanz, Stephan Irsen, Marc Beyer, Joachim L. Schultze, Martin J. Dyer, Paolo Salomoni, Dan Ehninger, Pierluigi Nicotera, Daniele Bano

**Affiliations:** 1 German Center for Neurodegenerative Diseases (DZNE), Bonn, Germany; 2 Center of Advanced European Studies and Research (caesar), Bonn, Germany; 3 Ernest and Helen Scott Haematological Research Institute, University of Leicester, Leicester, UK

## Abstract

Chromatin remodelers have emerged as prominent regulators of epigenetic processes and potential drivers of various human pathologies. The multi-subunit chromatin-remodeling SWI/SNF complex determines gene expression programs and, consequently, contributes to the differentiation, maturation and plasticity of neurons. Here, we investigate the elusive biological function of Bcl7a and Bcl7b, two newly identified subunits of the SWI/SNF complex that are highly expressed throughout the brain. We generated ubiquitous and neuron-specific Bcl7a and Bcl7b single and double knockout mice. We provide evidence that Bcl7b is dispensable for animal survival as well as behavioral plasticity. Conversely, ubiquitous Bcl7a knockout results in perinatal lethality, while genetic deletion of *Bcl7a* in postmitotic neurons elicits motor abnormalities and affects dendritic branching of Purkinje cells, with no obvious synergistic relationship with Bcl7b. Collectively, our findings reveal novel insights into the cellular processes linked to BCL7-containing SWI/SNF complexes and their unrecognized roles in the brain.

## Introduction

Chromatin structure regulates the accessibility to the genetic material. Multiple expression programs are established through the remodeling of chromatin components and the recruitment of the transcriptional machinery at target DNA sequences^1^. As a scaffolding unit of chromatin, each nucleosome comprises 147 bp of DNA coiled around particles of two copies each of histone H2A, H2B, H3 and H4.

Chromatin remodeling complexes modulate the chromatin status primarily by affecting histone-DNA interactions. The SWItch/Sucrose Non-Fermenting (SWI/SNF) complex is one of the ATP-dependent chromatin remodelers in eukaryotic cells. Initially characterized in *Saccharomyces cerevisiae* ^2,3^, the purified yeast SWI/SNF is a large multi-subunit complex of approximately 1.1 MDa and consists of eleven distinct polypeptides^4–6^. The catalytic core subunit Swi2/Snf2 has an ATP-dependent helicase activity^7^, while the other elements possess regulatory domains that control enzymatic activity, facilitate the binding to chromatin and confer functional specificity to the complex^4,5,8^. Following ATP hydrolysis, the SWI/SNF complex efficiently stimulates chromatin remodeling through the displacement and eviction of histones and exposure of nucleosomal DNA^9–11^. Ultimately, the yeast SWI/SNF dictates the expression of a large subset of inducible genes through the recruitment of the transcriptional machinery

The composition of SWI/SNF complexes underwent significant changes during metazoan evolution. While the yeast SWI/SNF complex positively enhances transcriptional activity in yeast, mammalian SWI/SNF complexes can both repress or activate target genes. As a response to the increased chromatin organization of multicellular organisms, SWI/SNF complexes gained and replaced distinct components, thus acquiring specialized activity and selectivity towards targeted genes. Contrary to its yeast counterpart, mammalian SWI/SNF complexes, also known as BAF (Brg/Brahma-associated factors) complexes, are heterogeneous and incorporate cell-type and developmental-specific subunits. The two mutually exclusive ATPases, SMARCA4/BRG1 (Brahma-related gene 1) and SMARCA2/BRM (Brahma), associate with various factors. The 2MDa polymorphic arrays include the five yeast homologs plus novel associated subunits gained during the course of evolution^8,12^. Although isolated BRG1 and BRM are sufficient to promote ATP-dependent reactions, the reconstitution of the complex with the conserved subunits further stimulates nucleosome shift *in-vitro* ^13^. In mammalian cells, the functional core unit consists of one of the two highly homologous BRG1 and BRM enzymes, together with BAF250a/b, BAF170/BAF155, BAF60a/b/c, BAF57, BAF53a/b, BAF47 and actin^12,14,15^. New additional non-exchangeable components of the SWI/SNF complexes were recently identified using proteomic-based approaches^16–18^, considerably increasing the number of theoretical combinatorial assemblies with potential biological significance^12^. As a distinct feature, mammalian SWI/SNF complexes seem to perform very specialized functions and critically control cell lineage specification.

Given its importance in stemness and cell lineage commitment, it is perhaps not surprising that either inherited or arising *de novo* mutations have been associated with human pathologies, with nearly 20% of all human cancers harbouring a mutation in at least one of the SWI/SNF subunits^17,19^. One unifying interpretation is that aberrant residual SWI/SNF assemblies drive oncogenic processes through a dysregulated transcriptional state of the cell^19,20^. Besides its tumor suppressor function, dominant mutations of SWI/SNF components have been also implicated in severe intellectual disabilities, including Coffin-Siris and Nicolaides-Baraitser syndromes^21–26^. Thus, altered SWI/SNF complex function seems to be causally linked to neurologic disorders in humans. In line with this possibility, a wealth of recent work in model organisms supports a fundamental role of the SWI/SNF complex in neurodevelopment and cognition (reviewed in^12,27,28^). In the nervous system, the tightly regulated recruitment of specific SWI/SNF assemblies allows for the engagement of transcriptional programs that ultimately results in diversified expression patterns. Consistently, the precise composition of these assemblies endows pluripotency of embryonic stem cells, while the exchange of certain dedicated subunits induces the transition to terminally differentiated neurons, further highlighting the little biological redundancy of these complexes^12,29,30^. Despite the relevance of these biological aspects, the importance of individual SWI/SNF complexes in animal behavior and brain function has been only marginally addressed.

The B-cell lymphoma/leukemia protein 7 A (or B-cell CLL/lymphoma 7 A protein) was initially cloned from a chromosomal translocation in a Burkitt lymphoma cell line^31^. The BCL7-family members possess a distinctive N-terminal domain evolutionarily conserved throughout the animal kingdom, whereas there is little homology between family members in the remaining sequence^32^. This might result in functional divergences of the isoforms. Through independent proteomic approaches, the three human BCL7 proteins were purified as novel, non-exchangeable subunits of the SWI/SNF complex, in a stoichiometry very similar to other well-established components^16–18^. Prior studies in invertebrates suggest a role of BCL7-like proteins as a transcriptional regulatory component of WNT/Wingless and Notch signaling pathways^33,34^. Yet, our knowledge on the molecular function of BCL7 proteins lags behind. Here, we sought to determine the importance of Bcl7-containing SWI/SNF complexes in the brain. As both *Bcl7a* and *Bcl7b* are highly expressed throughout the mouse brain, we generated conditional single and double knockout mice. We show that neuron-specific Bcl7a deficiency alters the highly branched dendritic trees of cerebellar Purkinje cells and impairs motor skills, whereas Bcl7b knockout has no obvious effect on animal survival and behavior. Taken together, our findings describe the contribution of Bcl7a and Bcl7b in mouse survival and brain physiology.

## Results

### Bcl7a, but not Bcl7b, is required for mouse survival

**Figure 1:**
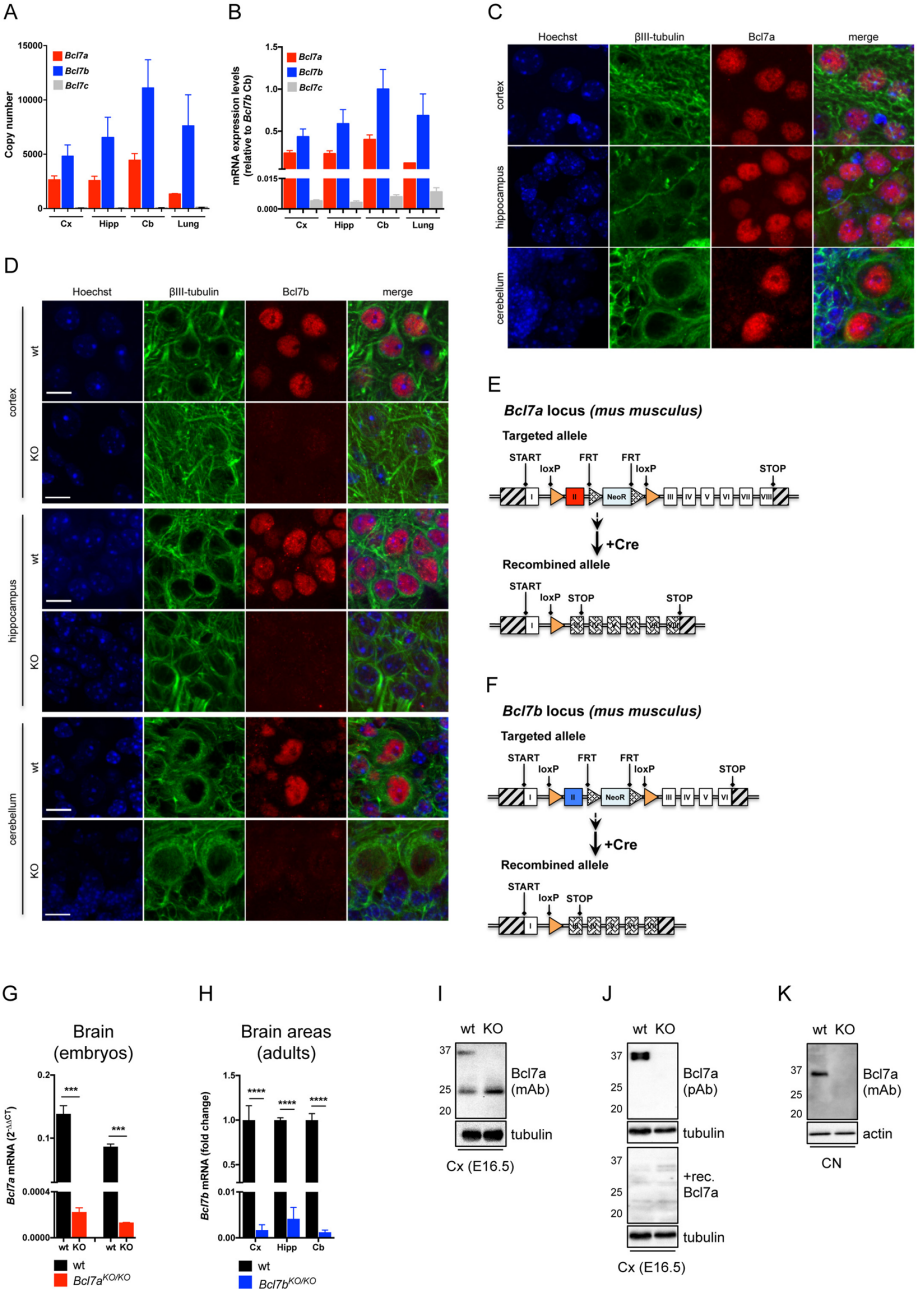
Bcl7a and Bcl7b are highly expressed in the mouse brain. (**A**) Absolute quantitative Real Time PCR analysis of *Bcl7a/b/c* copy number/μl in adult mouse tissues (Cx = cortex; Hipp = hippocampus; Cb = cerebellum). (**B**) qRT-PCR of *Bcl7a/b/c* mRNA expression levels in adult mouse tissues (relative to *Bcl7b* in Cb). (**C**) Immunofluorescence staining of Bcl7a in the adult mouse brain. Panels show the high expression and nuclear localization of Bcl7a immunoreactivity (red). β-III tubulin (green) was used as neuronal marker. Hoechst-33342 was used to stain nuclei (blue). (**D**) Immunofluorescence staining of Bcl7b (red) in adult wild type (wt) and *Bcl7b* ^*KO/KO*^ brains. Panels show the predominant nuclear localization of Bcl7b immunoreactivity in wt brains, which is absent in *Bcl7b* ^*KO/KO*^ mice. β-III tubulin (green) was used as neuronal marker. Scale bar = 20 μm. (**E**,**F**) Schematic representations of (**E**) *Bcl7a* and (**F**) *Bcl7b* floxed alleles. Cre-mediated recombination results in the knockout of the floxed alleles. (**G**) qRT-PCR of *Bcl7a* mRNA levels in embryonic brains (E18.5) from wild type (n = 2) and full *Bcl7a* ^*KO/KO*^ (n = 2) littermates (values are 2^−ΔΔCT^; mean ± S.E.M.; ***p < 0.001). (**H**) qRT-PCR of *Bcl7b* mRNA levels in adult brains from wild type and *Bcl7b* ^*KO/KO*^ littermates (Cx = cortex; Hipp = hippocampus; Cb = cerebellum; mean ± S.E.M.; ****p < 0.0001). (**I**) Immunoblot analysis of Bcl7a expression levels in the cortex (Cx) of wt and *Bcl7a* ^*KO/KO*^ embryos (E16.5). (**J**) Pre-absorption with recombinant Bcl7a protein ablates immunoreactivity of Bcl7a antibody. (**K**) Immunoblot analysis of Bcl7a protein expression in wt and Bcl7a KO primary cortical neurons (CN). Uncropped blots are provided as Supplementary Information.

Since distinct chromatin remodelers regulate neural stem cell states, neuronal differentiation and plasticity^8,28,29^, we sought to characterize the importance of Bcl7 isoforms in the mouse brain. As a first step, we tested whether the three *Bcl7* genes are expressed in the central nervous system. We performed quantitative Real-Time PCR (qRT-PCR) in samples from different mouse brain areas and lung (as a non-neuronal tissue). We detected *Bcl7a/b/c* mRNA in all the analyzed tissues, albeit *Bcl7a* and *Bcl7b* were much more expressed compared to *Bcl7c*, reaching an above 100-fold difference in the cerebellum (Fig. 1A,B). Both *Bcl7a* and *Bcl7b* mRNAs were detected in neuronal and glial cells (data not shown). Next, we assessed the expression and subcellular localization of Bcl7a and Bcl7b proteins. We found a clear nuclear staining in various areas of brains from postnatal and adult animals (Fig. 1C,D and Supplemental Figure S1A,B). Since *Bcl7a* and *Bcl7b* are abundantly expressed in the nervous system, we generated transgenic mice carrying floxed *Bcl7a*, *Bcl7b* and *Bcl7a; Bcl7b* alleles (Fig. 1E,F). Mice harboring either *Bcl7a* or *Bcl7b* floxed alleles were crossed with a line ubiquitously expressing the bacterial Cre-recombinase (i.e., CMV-Cre^tg/wt^). While Bcl7b full knockout mice were obtained in a Mendelian manner (data not shown), ubiquitous Cre-mediated deletion of *Bcl7a* resulted in viable E16.5 embryos at almost expected distribution, however only approximately 4% of Bcl7a deficient pups survived until weaning and into adulthood (Supplemental Figure S1C). We regularly surveyed pregnant females until parturition and recovered only a few partially consumed carcasses. Histological analyses of Bcl7a-deficient offspring as well as of E19.5-E20.5 embryos showed no obvious abnormalities. We speculate that newborn *Bcl7a* ^*KO/KO*^ pups fail to initiate normal respiration or to feed properly. To validate the successful Cre-mediated recombination of the floxed alleles, we performed qRT-PCR and showed the impaired expression of *Bcl7a* and *Bcl7b* mRNAs in the respective knockout animals (Fig. 1G,H). Consistent with our gene targeting strategy, Cre-mediated ablation caused the loss of Bcl7b (Fig. 1D and Supplemental Figure S1B) and Bcl7a proteins (Fig. 1I,K). Of note, a monoclonal antibody recognized a 25 kDa band in immonoblots of tissue from both wild type and knockout brains (Fig. 1I), although this band was absent in both wt and Bcl7a KO primary cortical neurons (Fig. 1K). Based on our antibody validation (unpublished data) and pre-absorption experiment (Fig. 1J), we can assert that the band is non-specific. Conversely, none of the tested Bcl7b antibodies were specific in immunoblot experiments (data not shown). In summary, our findings indicate that Bcl7a, but not Bcl7b, is required for mouse survival.

### Genetic ablation of *Bcl7b* does not compromise animal behavior

**Figure 2:**
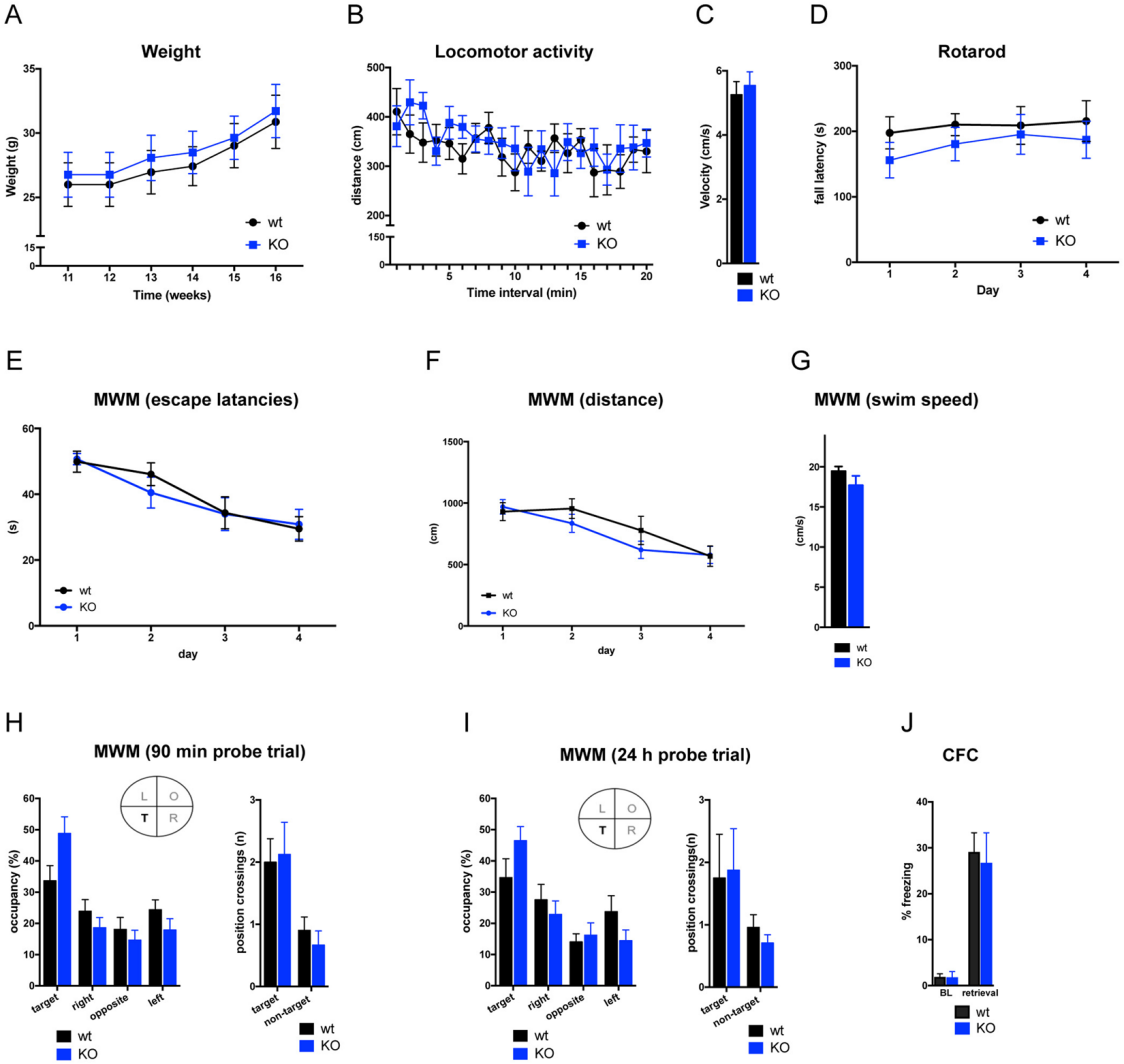
Genetic ablation of *Bcl7b* does not impair motor coordination or cognitive function. (**A**) No body weight differences were seen between *Bcl7b* ^*KO/KO*^ (KO) and wild type (wt) mice (wt: n = 8 (5 ♂, 3 ♀); KO: n = 8 (5 ♂, 3 ♀)). ANOVA: effect of week F_5,70_ = 59.64, *p* < 0.0001; effect of genotype: F_1,14_ = 0.1237, *p* = 0.7303). (**B**) Locomotor activity levels and (**C**) average movement velocity during the open field test were similar in *Bcl7b* ^*KO/KO*^ (n = 8; 5 ♂, 3 ♀) and control (n = 8; 5 ♂, 3 ♀) mice (ANOVA: effect of time interval: F_19,266_ = 2.744, *p* = 0.0002; effect of genotype: F_1,14_ = 0.1142, *p* = 0.7404). (**D**) Fall latencies on the accelerating rotarod did not differ between genotypes, indicating that motor coordination and balance were unaltered in *Bcl7b* ^*KO/KO*^ animals (wt: n = 8 (5 ♂, 3 ♀), KO: n = 8 (5 ♂, 3 ♀)) (ANOVA: effect of day: F_3,42_ = 1.086, *p* = 0.3656; effect of genotype: F_1,14_ = 0.7618 *p* = 0.3975). (**E**–**G**) Acquisition training in the Morris water maze (MWM). (**E**) Escape latencies and (**F**) swim distances gradually decreased over the four days of training (ANOVA: effect of day F_3, 42_ = 11.73 *p* < 0.0001) with no differences between *Bcl7b* ^*KO/KO*^ (n = 8; 5 ♂, 3 ♀) and wild type (n = 8; 5 ♂, 3 ♀) mice (ANOVA: effect of genotype F_1,14_ = 0.0788, *p* = 0.783). (**G**) The average swim speed over all training trials was comparable between genotypes (*t* test *p* = 0.2018). (**H**,**I**) MWM probe trials with quadrant occupancy and platform position crossings used as index for spatial memory function. No effect of genotype was seen during (**H**) short-term (90 min delay) and (**I**) long-term (24 h delay) memory probe trials as *Bcl7b* ^*KO/KO*^ and wild type mice showed a similar preference for the target quadrant (ANOVA: effect of quadrant F_3,39_ = 11.66 *p* < 0.0001 and F_3,42_ = 9.388, *p* = 0.0001; effect of genotype F_1,13_ = 0.5115 *p* = 0.4871 and F_1,14_ = 0.2835 *p* = 0.6028) as well as comparable numbers of target position crossings (T = target, R = right, L = left, O = opposite; wt: n = 7 (4 ♂, 3 ♀), KO: n = 8 (5 ♂, 3 ♀)). (**J**) In the contextual fear conditioning (CFC) task, no differences were seen regarding baseline (BL) freezing levels between *Bcl7b* ^*KO/KO*^ (n = 8; 5 ♂, 3 ♀) and control (n = 8; 5 ♂, 3 ♀) mice. During the retrieval trial, performed 24 h after CFC training, *Bcl7b* ^*KO/KO*^ and control mice showed similar levels of freezing, indicating normal long-term memory function (*t* test *p* = 0.3473). Data are presented as mean ± S.E.M.

To gain insights into the biology of Bcl7-family members, first we assessed animals lacking Bcl7b, given its high expression levels in the adult mouse brain. In general, *Bcl7b* ^*KO/KO*^ animals developed normally and did not differ from control littermates regarding body weight (Fig. 2A) and the overall brain morphology (data not shown). Behavioral experiments were performed with wild type and *Bcl7b* ^*KO/KO*^ male and female mice. Since we did not detect major sex differences in any of the behavioral parameters tested (Supplementary Figure S2A–E and Supplementary Table 1), those data were pooled for the respective genotypes. In the open field test, *Bcl7b* ^*KO/KO*^ animals showed no alterations in locomotor activity levels or exploratory behavior (Fig. 2B,C). Also, loss of Bcl7b did not lead to motor coordination deficits as assessed via the rotarod test (Fig. 2D). When tested in the Morris water maze (MWM), a well-established test for spatial learning and memory, *Bcl7b* ^*KO/KO*^ mice performed similar to wild type animals in terms of swim speed, gradually decreasing escape latencies and swim distances (Fig. 2E,G). Likewise, no differences were seen in short- and long-term memory probe trials since both genotypes showed a similar preference for the target quadrant as well as comparable numbers of platform position crossings (Fig. 2H,I). As another hippocampus-dependent task, we then assessed contextual fear conditioning (CFC). Here, *Bcl7b* ^*KO/KO*^ mice did not differ from control littermates regarding contextual fear memory retrieval assessed 24 h after the conditioning trial (Fig. 2J). Based on our first set of data, it seems that Bcl7b does not severely compromise motor coordination and memory formation.

### Bcl7a contributes to motor coordination

**Figure 3:**
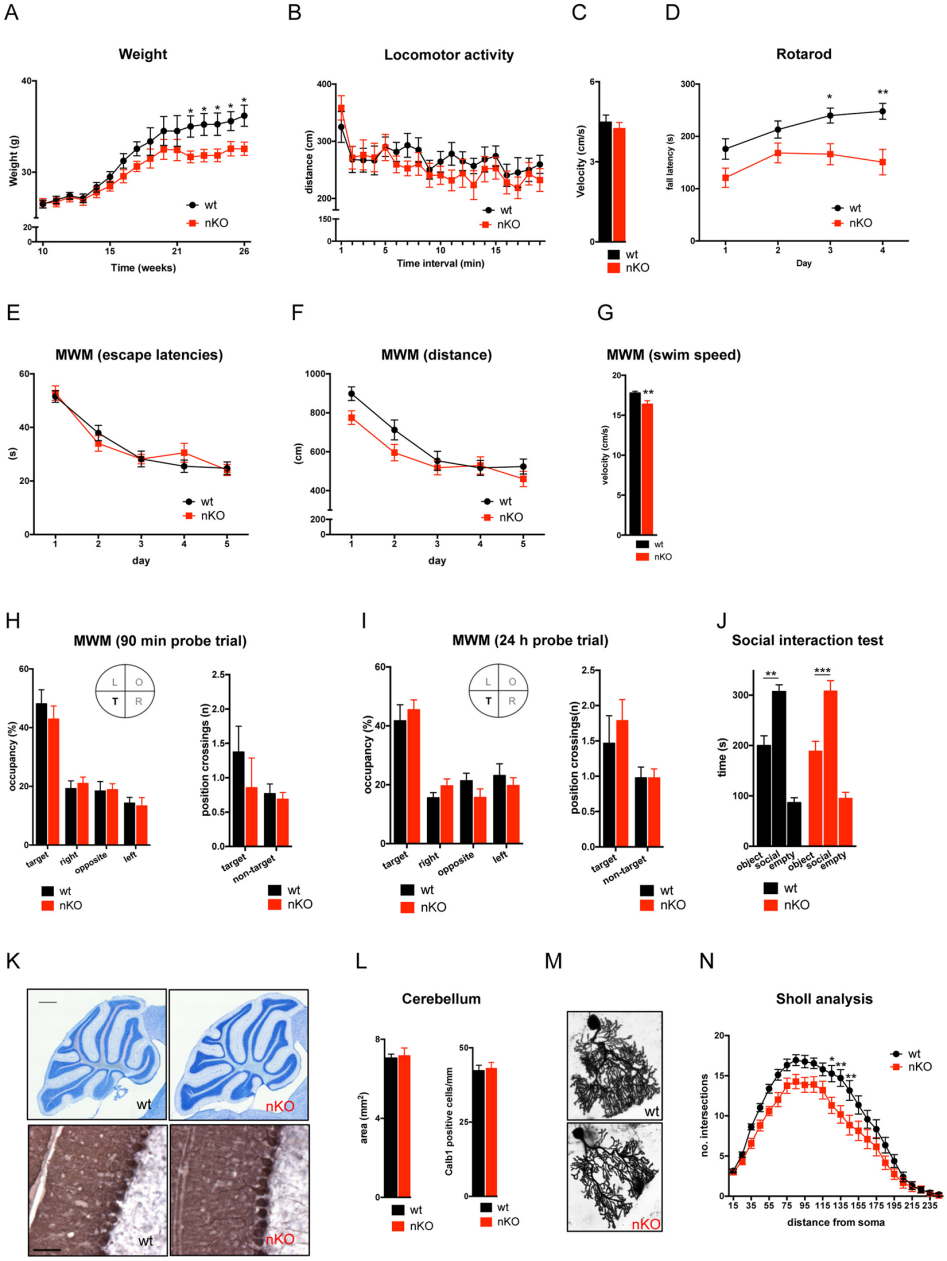
Neuron-specific deletion of *Bcl7a* affects motor coordination and Purkinje cell arborization. (**A**) Body weight changes from 10 to 27 weeks of age. Bcl7a^*nKO*^ mice show a slight reduction in weight gain around 6 months of age (wt: n = 16; nKO: n = 14). (**B–C**) During the open field test, (**B**) locomotor activity levels and (**C**) the average movement velocity were similar in *Bcl7a* ^*nKO*^ (nKO, n = 14) and wild type (wt, n = 16) littermates. (**D**) Compared to littermate controls (n = 16), *Bcl7a* ^*nKO*^ mice (n = 14) failed to improve their performance on the accelerating rotarod (ANOVA: effect of genotype F_1,28_ = 8.999, *p* = 0.0056 and day F_3,84_ = 11.15, *p* < 0.001), resulting in significantly lower fall latencies on day 3 (Bonferroni post hoc *t* test *p* = 0.0216) and day 4 (Bonferroni post hoc *t* test *p* = 0.0012). (**E**–**G**) Acquisition training in the Morris water maze (MWM). (**E**) Escape latencies and (**F**) swim distances gradually decreased over the five days of training (ANOVA: effect of day F_4,112_ = 37.51, *p* < 0.0001 and F_4,112_ = 23.43, *p* < 0.0001, respectively) which was similar in *Bcl7a* ^*nKO*^ (n = 14) and control littermates (n = 16) as indexed by a lack of a significant effect of genotype. (**G**) The average swim speed during training trials was significantly lower in *Bcl7a* ^*nKO*^ mice (*t* test *p* = 0.0088). (**H**,**I**) MWM probe trials with quadrant occupancy and platform position crossings as parameters for spatial memory. No significant effects of genotype were seen in (**H**) short-term (90 min delay) and (**I**) long-term (24 h delay) memory probe trials as *Bcl7a* ^*nKO*^ (n = 14) and wild type (n = 16) mice showed a similar preference for the target quadrant (ANOVA: effect of quadrant F_3,84_ = 30, *p* < 0.0001 and F_3,81_ = 20.84, *p* < 0.0001) as well as comparable numbers of platform position crossings. (**J**) Three-chamber social interaction test showing the mean duration of time spent in each chamber. There was a significant effect of chamber (ANOVA F_2,40_ = 54.4, *p* = 0.001) but no effect of genotype, and the preference for the social over the object compartment was similar in *Bcl7a* ^*nKO*^ (n = 12, Bonferroni post hoc *t* test *p* = 0.0004) and control animals (n = 10, Bonferroni post hoc *t* test *p* = 0.0036). (**K**) Nissl staining and DAB immunohistochemistry of calbindin-labeled Purkinje cells in sagittal brain sections did not reveal morphological alterations in *Bcl7a* ^*nKO*^ mice. Scale bar upper images: 500 μm, scale bar lower images: 100 μm. (**L**) Quantification of the cerebellar size and the number calbindin (Calb1)-positive cells within the cerebellum of wild type control (wt, n = 5) and *Bcl7a* ^*nKO*^ (nKO, n = 4) mice. (**M**) Representative images of Golgi-stained Purkinje cells from adult wild type and *Bcl7a* ^*nKO*^ mice. (**N**) Sholl analysis of the dendritic arbor of Purkinje neurons. Branch intersections were quantified at 10 μm-spaced concentric circles starting from the cell soma. The dendritic complexity of Purkinje cells was significantly altered in *Bcl7a* ^*nKO*^ mice as indexed by marked effects of genotype (ANOVA: F_1,90_ = 117.6, *p* < 0.0001) and a genotype x radius interaction (ANOVA: F_23,2070_ = 1.864, *p* = 0.0076), showing reduced numbers of intersections at 125 μm (Bonferroni post hoc *t* test *p* = 0.0128), 135 μm (Bonferroni post hoc *t* test *p* = 0.0017) and 145 μm (Bonferroni post hoc *t* test *p* = 0.0038) distance from the cell soma (wt: n = 53: nKO: n = 39). Data are presented as mean ± S.E.M.; asterisks denote significant differences (*p < 0.05, **p < 0.01, ***p < 0.001).

To overcome the limitation due to its lethal phenotype, we generated tissue-specific Bcl7a knockouts. Notably, we did not use mice carrying the Nestin-Cre transgene because they often display aberrant metabolic profiles as well as behavioral defects^35^. Thus, *Bcl7a* ^*fl/fl*^ males were bred with females expressing the Cre-recombinase under the neuronal-specific Synapsin promoter (*Syn-Cre* ^*tg/wt*^). This strategy allows for the specific deletion *Bcl7a* floxed allele in a large number of postmitotic neurons in the brain, albeit it may partially result in cellular mosaicism due to the intrinsic variability of the Cre-lox system^35,36^. We obtained *Bcl7a* ^*fl/fl*^;*Syn-Cre* ^*tg/wt*^ (herein referred to as *Bcl7a* ^*nKO*^) at expected ratios and with normal appearance at weaning. The loss of *Bcl7a* expression was confirmed via qRT-PCR across different brain regions (Supplemental Figure S3A). In line with previously published observations^36^, Synapsin-Cre-driven recombination occurred in most, but not all, neurons, as shown by immunohistochemistry in the hippocampus and cerebellum of *Bcl7a* ^*nKO*^ mice (Supplemental Figure S3B– C). Overall, *Bcl7a* ^*nKO*^ animals developed normally, although a decrease in body weight was observed at approximately 6 months of age (Fig. 3A). We assessed common behavioral traits of *Bcl7a* ^*nKO*^ animals. Adult *Bcl7a* ^*nKO*^ mice did not differ from control littermates in the open-field test indicating that general locomotor activity levels, exploratory and anxiety-like behaviors were normal (Fig. 3B,C). However, *Bcl7a* ^*nKO*^ animals showed a slightly altered gait, as assessed via the catwalk test, which was indexed by an increased front paw stand duration and stride length as well as a reduced paw overlap (Supplemental Figure S3D–G: *p* < 0.05 for all parameters). Furthermore, *Bcl7a* ^*nKO*^ mice did not improve their performance on the rotarod and showed lower fall latencies compared to control littermates (Fig. 3D: *p* < 0.05 for day 3 and day 4). Overall, these data suggest that neuron-specific *Bcl7a* knockout causes motor coordination deficits. Next, adult wild type and *Bcl7a* ^*nKO*^ mice were tested for short- and long-term memory performance in the MWM. Over the five days of training, escape latencies and swim distances to reach the platform gradually decreased in a similar manner for wild type and *Bcl7a* ^*nKO*^ littermates, indicating that spatial learning was unaltered in *Bcl7a* ^*nKO*^ mice (Fig. 3E,F). However, *Bcl7a* ^*nKO*^ animals showed a significant reduction in the average swim speed (Fig. 3G: *p* < 0.01) as well as slightly increased floating behavior (data not shown), most likely due to the motor coordination deficits described earlier. Short-term memory performance was tested on day three 90 min after the last block of training trials, while long-term memory performance was assessed 24 h after the last training session. In both tests, the time spent in the target quadrant was comparable between wild type and *Bcl7a* ^*nKO*^ animals. Likewise, there were also no differences in the number of platform position crossings between the two genotypes (Fig. 3H,I). These data indicate that *Bcl7a* ^*nKO*^ mice and control littermates had learned the task and recalled the platform position equally well. Additionally, we assessed social behavior in the three-chamber social preference test but did not detect any differences between *Bcl7a* ^*nKO*^ and control mice as both genotypes showed a similar preference for the social over the object compartment (Fig. 3J).

Given the impaired performance of *Bcl7a* ^*nKO*^ mice in the rotarod test (Fig. 3D) and the negligible effect on learning paradigms (Fig. 3E,F), we focused on neuroanatomical aspects of the cerebellum, a brain region that has been crucially linked to motor coordination. Foliation and lamination of the cerebellar cortex as well as the cerebella size appeared normal in *Bcl7a* ^*nKO*^ mice as assessed by histological analysis of sagittal brain sections (Fig. 3K,L). Furthermore, immunostaining of Purkinje cells did not reveal any differences in either the number or the alignment of Purkinje neurons (Fig. 3K). We then analyzed the morphology of Purkinje cell dendrites using Golgi-stained cerebellar sections (Fig. 3M). Here, the complexity of the dendritic arbor as assessed by Sholl analysis was significantly reduced in *Bcl7a* ^*nKO*^ animals (Fig. 3N: *p* < 0.001). Taken together, these findings suggest that neuron-specific Bcl7a deficiency does not lead to memory impairments, while it affects the dendritic branching of Purkinje cells and leads to motor dysfunction.

### Bcl7b deficiency does not further exacerbate Bcl7a-dependent neuronal dysfunction

**Figure 4:**
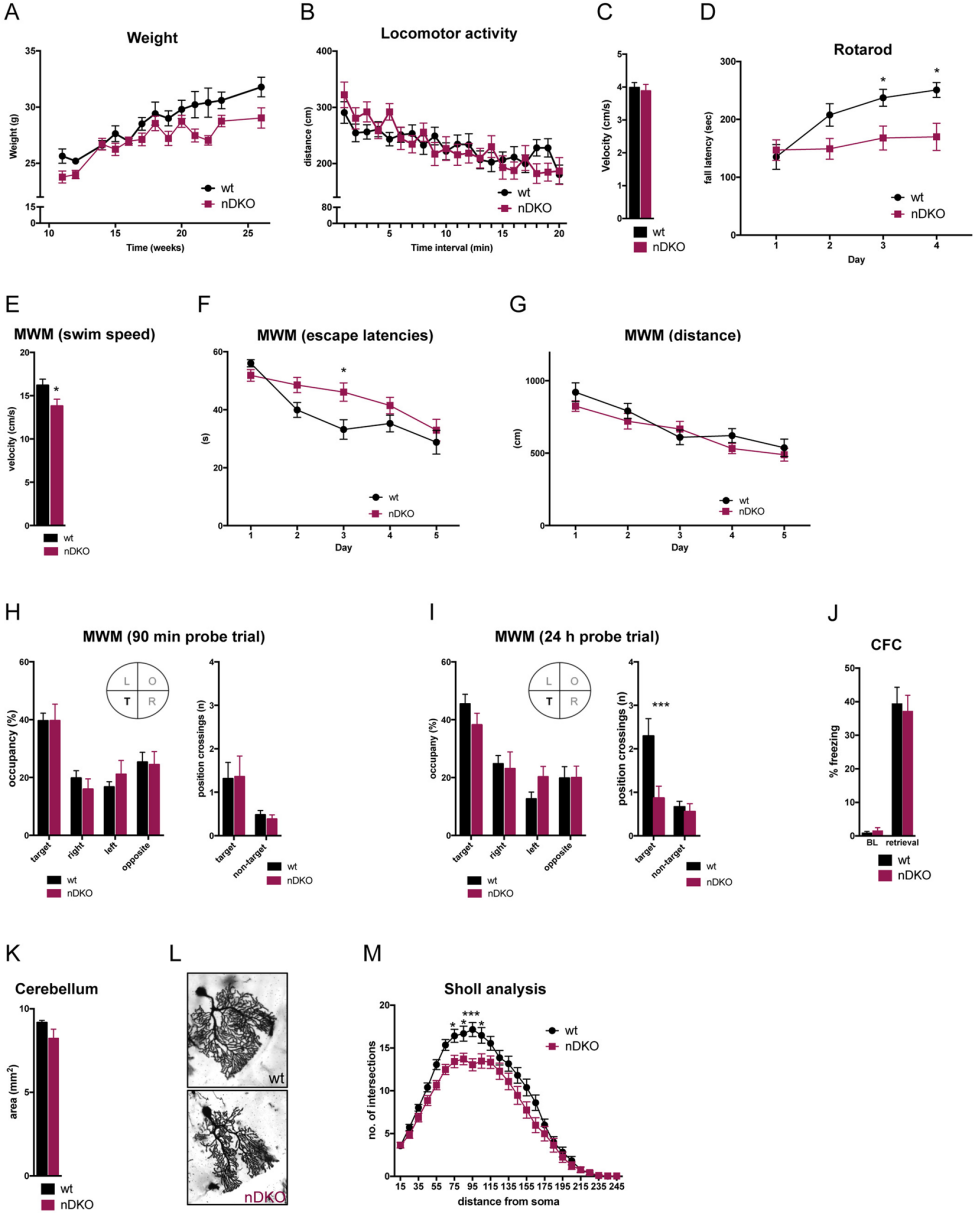
Genetic deletion of *Bcl7b* does not further exacerbate Bcl7a-dependent neuronal dysfunction. (**A**) *Bcl7* ^*nDKO*^ mice had a slight reduction in body weight gain compared to wild type (wt) littermates (wt: n = 18; nDKO: n = 15). (**B**) Locomotor activity levels and (**C**) average movement velocity during the open field test did not differ between *Bcl7* ^*nDKO*^ (n = 15) and wild type (n = 18) animals. (**D**) On the accelerating rotarod, *Bcl7* ^*nDKO*^ mice (n = 11) showed no performance improvement over the four days of training, contrary to control littermates (n = 11) (ANOVA: effect of day F_3,60_ = 10.77, *p* < 0.0001; effect of genotype F_1,20_ = 5.564, *p* = 0.0286; day x genotype interaction F_3, 60_ = 4.845, *p* = 0.0044). These motor coordination impairments resulted in significantly lower fall latencies on training day 3 (Bonferroni post hoc *t* test *p* = 0.043) and day 4 (Bonferroni post hoc *t* test *p* = 0.0125) compared to wild type animals. (**E**–**G**) Acquisition training in the Morris water maze (MWM). (**E**) Over all training trials, the average swim speed was significantly reduced in *Bcl7* ^*nDKO*^ (n = 15) compared wild type (n = 17) mice. (**F**) In comparison to control mice, escape latencies during MWM training improved slightly slower in *Bcl7* ^*nDKO*^ animals (ANOVA: effect of day F_4,120_ = 22.77, *p* < 0.0001; day x genotype interaction F_4,120_ = 3.162, *p* = 0.0165), which was most pronounced on training day 3 (Bonferroni post hoc *t* test *p* = 0.013). (**G**) However, the gradual decrease of swim distances was similar in both genotypes (ANOVA: effect of day F_4,120_ = 18.87, *p* < 0.0001), suggesting that spatial learning was not impaired in *Bcl7* ^*nDKO*^ mice. (**H**,**I**) MWM probe trials with quadrant occupancy and platform position crossings used as measures for spatial memory. No effect of genotype was seen in (**H**) short-term (90 min delay) and (**I**) long-term (24 h delay) memory probe trials regarding quadrant occupancies as *Bcl7* ^*nDKO*^ (n = 14–15) and wild type mice showed similar target quadrant preferences (ANOVA: F_3,81_ = 9.903, *p* < 0.0001 and F_3,90_ = 13.33, *p* < 0.001). While there were no genotype differences in the number of target position crossings during the short-term memory probe trial, *Bcl7* ^*nDKO*^ mice showed a significantly lower number of target crossings in the long-term memory test (ANOVA: effect of position F_1, 30_ = 12.16, *p* = 0.0015; effect of genotype F_1,30_ = 8.366, *p* = 0.0071, position x genotype interaction F_1,30_ = 5.609, *p* = 0.0245; Bonferroni post hoc *t* test *p* = 0.0009; T = target, R = right, L = left, O = opposite). (**J**) In the contextual fear conditioning (CFC) paradigm, no differences between *Bcl7* ^*nDKO*^ (n = 15) and control mice (n = 18) were seen regarding baseline (BL) freezing levels. During the retrieval trial, performed 24 h after CFC training, freezing levels did not differ between *Bcl7* ^*nDKO*^ (n = 15) and control mice (n = 18), indicating normal long-term memory function. (K) No differences were seen in the cerebellar size of adult *Bcl7* ^*nDKO*^ (n = 3) and wild type (n = 3) mice. (**L**) Representative images of Golgi-stained Purkinje neurons from *Bcl7* ^*nDKO*^ and wild type brains. (**M**) Sholl analysis of the dendritic arborization of Purkinje neurons. Branch intersections were quantified at 10 μm-spaced concentric circles starting from the cell soma. The dendritic complexity of Purkinje cells was significantly altered in *Bcl7* ^*nDKO*^ mice as indexed by marked effects of genotype (ANOVA: F_1,91_ = 11.93, *p* = 0.0008) and a genotype x radius interaction (ANOVA: F_24,2184_ = 2.213, *p* = 0.0006), showing reduced numbers of intersections at 75 μm (Bonferroni post hoc *t* test *p* = 0.0344), 85 μm (Bonferroni post hoc *t* test *p* = 0.0402), 95 μm (Bonferroni post hoc *t* test *p* = 0.0003) and 105 μm (Bonferroni post hoc *t* test *p* = 0.0365) distance from the cell soma (wt: n = 53: nDKO: n = 40). Data are presented as mean ± S.E.M.; asterisks denote significant differences (*p < 0.05, **p < 0.01, ***p < 0.0001).

Since *Bcl7a* and *Bcl7b* are the two dominant isoforms in the nervous system (Fig. 1A,B), we hypothesised that compensatory processes may be present in single knockout animals, explaining the mild phenotypes observed in our knockout mice. To test this possibility, we crossed *Bcl7a* ^*fl/fl*^ *;Bcl7b* ^*fl/fl*^ males with *Syn-Cre* ^*tg/wt*^ females and obtained neuron-specific *Bcl7a*;*Bcl7b* double knockout (herein referred to as *Bcl7* ^*nDKO*^) animals. Cre-mediated recombination of *Bcl7a* and *Bcl7b* floxed alleles was confirmed via qRT-PCR and immunostainings in various brain areas of *Bcl7* ^*nDKO*^ mice (Supplemental Figure S4A–D). Overall, *Bcl7* ^*nDKO*^ mice showed normal development with a mild reduction in body weight (Fig. 4A) and no obvious changes in brain morphology (not shown). Similar to *Bcl7a* ^*nKO*^ animals, *Bcl7* ^*nDKO*^ mice did not differ from control littermates regarding locomotor activity and velocity in the open field (Fig. 4B,C), although they displayed significant motor coordination impairments on the rotarod (Fig. 4D: *p* < 0.05). Next, we tested spatial learning and memory in the MWM. As observed in *Bcl7a* ^*nKO*^ animals, *Bcl7* ^*nDKO*^ mice showed a significant reduction in the average swim speed (Fig. 4E: *p* < 0.05) as well as higher levels of floating behavior (data not shown) compared to control littermates. This inability to swim properly subsequently resulted in a slower improvement of escape latencies during training trials (Fig. 4F), while the gradual decrease of swim distances was comparable between control and *Bcl7* ^*nDKO*^ mice (Fig. 4G), suggesting that spatial learning is intact. Short- and long-term memory performance was assessed on day 3 and day 6, respectively. In both tests, the time spent in the target quadrant was comparable between the two genotypes (Fig. 4H,I), while *Bcl7* ^*nDKO*^ mice showed a clear reduction of platform position crossings in the 24 h probe trial (Fig. 4I: wt vs. *Bcl7* ^*nDKO*^ *p* < 0.001). However, this effect is most likely due to impaired motor function and increased floating behavior rather than indexing a spatial memory deficit in recalling the previously learned platform location. To support this possibility, we next performed contextual fear conditioning. Here, *Bcl7* ^*nDKO*^ displayed similar levels of freezing during fear memory retrieval trials (Fig. 4J), ruling out further any memory-related deficits in *Bcl7* ^*nDKO*^ mice. Since the motor coordination impairments of *Bcl7* ^*nDKO*^ mice phenocopy those observed in *Bcl7a* ^*nKO*^ animals, we again assessed different aspects of cerebellar morphology using histological staining of mid-sagittal brain sections. As in *Bcl7a* ^*nKO*^ mice, *Bcl7* ^*nDKO*^ animals did not show alterations in the cerebellar size or the foliation and lamination of the cerebellar cortex (Fig. 4K). Likewise, we did not detect any changes in Purkinje cell density or alignment (data not shown), whereas Sholl analysis of Golgi-impregnated Purkinje cells revealed a significant reduction of the dendritic complexity in *Bcl7* ^*nDKO*^ mice (Fig. 4L,M: *p* < 0.001). In summary, *Bcl7* ^*nDKO*^ animals show a similar phenotype to *Bcl7a* ^*nKO*^ mice, indicating that Bcl7b plays no compensatory role in Bcl7a-dependent behaviors. Consistently, Bcl7b deficiency has no synergistic effects with neuron-specific *Bcl7a* knockout and does not exacerbate significantly the motor coordination impairments and reduced Purkinje cell complexity seen in *Bcl7a* ^*nKO*^ mice.

## Discussion

Chromatin remodelers have emerged as one of the contributors to neurodevelopmental disorders and intellectual disability syndromes in humans^12,27,28^. Previous studies in experimental models have further supported the importance of ATP-dependent chromatin remodelers in brain pathophysiology, demonstrating that their genetic manipulations cause a broad range of defects in the nervous system^37–43^. The SWI/SNF complex orchestrates the accessibility to well-defined chromatin territories, inducing unique expression patterns that critically govern neuronal identity and plasticity throughout the lifetime of an animal^12,28^. Multiple lines of evidence argue that the composition of the SWI/SNF complex critically determines the specific targeting to genomic sites^18,44–46^, probably through the recognition of distinct epigenetic marks and/or binding to certain transcription factors. In neurons, it was previously shown that BAF170 and BAF155 take part in brain development, since they influence Pax6-dependent neurogenesis and, consequently, cortical thickness^43,47^. Similarly, BAF53b is required for the correct targeting of the SWI/SNF complex to genes involved in dendritic outgrowth during development^42^. Mice expressing mutant Baf53b with a deletion of the C-terminal hydrophobic domain as well as *Baf53* ^*+/−*^ animals show deficits in hippocampus-dependent spatial long-term memory formation. These cognitive deficits were accompanied by altered long-term potentiation and abnormalities in dendritic spine structure such as a decreased ratio of thin- and mushroom-type spines^41,42^. Despite their relevance in biology and medicine, so far only a limited number of studies have addressed the contribution of individual SWI/SNF complex components in the maintenance and plasticity of postmitotic neurons. In this exciting context, we sought to unveil the role of Bcl7a and Bcl7b, two newly recognized subunits of the SWI/SNF complex with unknown biological function. First, we assessed their expression levels and subcellular localization in the mouse brain. We show that Bcl7a and Bcl7b are nuclear proteins that are abundantly expressed throughout different brain areas. Using a small sized animal cohort, we provide the first line of evidence that ubiquitous *Bcl7b* knockout does not considerably affect animal survival and behavioral function. Conversely, the ubiquitous deletion of *Bcl7a* leads to perinatal lethality, with very few *Bcl7a* ^*KO/KO*^ escapers surviving until adulthood, which seem to have a normal wild type phenotype. At the current stage, however, we cannot rule out the possibility that these animals are somatic mosaic mutants. Furthermore, we describe the tissue-specific contribution of Bcl7a and Bcl7b to motor coordination and cognitive function. We report that genetic deletion of *Bcl7a* alleles in postmitotic neurons causes motor coordination impairment as indicated by significantly lower fall latencies in the rotarod test. Much to our surprise, we did not observe any differences regarding spatial learning and memory formation in single and double knockout mice compared to control littermates. Although we cannot rule out that the impaired motor function seen following neuron-specific deletion of *Bcl7a* affects the analysis of some behavioral tests to a certain extend (e.g. the MWM), we believe that loss of Bcl7a and/or Bcl7b has no major effect on spatial learning and memory function *per se*. In support of this notion, no major differences were seen in the hippocampus-dependent contextual fear-conditioning paradigm, suggesting that spatial memory-associated processes are intact in *Bcl7a* ^*nKO*^ and B*cl7* ^*nDKO*^ mice.

As dendritic defects are common features of animals expressing mutated chromatin remodeling proteins^38,42,48^, we assessed the effect of Bcl7 deficiency on the dendritic complexity of Purkinje neurons, which are considered as the main output structure of the cerebellum. We found that neuron-specific Bcl7a knockout significantly reduced Purkinje cell arborization with no synergistic effects of Bcl7b deficiency, resembling the lack of additive responses on motor coordination following Bcl7a and Bcl7b loss. As recombination in Synapsin-Cre transgenic mice does not take place in neural progenitor cells, it seems that neuron-specific *Bcl7a* deletion has no effect on either the differentiation or the survival of cerebellar neurons. It may be that, upon neural commitment, Bcl7a becomes an auxiliary factor in certain neuronal subtypes. Future work is necessary to determine whether Bcl7a knockout in progenitor cells might lead to a more severe neuronal dysfunction in adult animals. Definitely, our neuron-specific single and double knockout animals show a relatively subtle phenotype compared to other chromatin remodeling mutants, such as *Snf2h* deficient mice, where Purkinje cells and granule neurons exhibit multiple defects that ultimately compromise their differentiation and survival^48^.

How does Bcl7a influence neuronal function? We have recently found that BCL7A knockout alters the expression of SWI/SNF complex subunits and compromises its binding to target transcriptional start sites in human tumorigenic cell lines (unpublished findings). Extending this concept to neurons, it is plausible to imagine that BCL7A knockout might affect gene transcription and, consequently, neuronal function because of diminished expression of, among others, BAF170 and BAF53, two SWI/SNF complex subunits that critically regulate neurogenesis and dendritic branching, respectively^41–43,47^. Our current data suggests that Purkinje neurons rely on Bcl7a for their correct development and/or maintenance. Given the limitation in terms of material and *in vitro* approaches, we attempted to recapitulate some of our findings in dissociated cortical and cerebellar granule neurons derived from control and knockout littermates. Disappointingly, in these cultured cells we could not identify any major phenotypic defects, including dendritic growth and branching as well as the response to excitatory stimuli (data not shown). Further work is required to understand how Bcl7a deficiency affects the SWI/SNF complex in a subset of cerebellar neurons (i.e., Purkinje cells).

Thus, although our descriptive work does not provide any molecular mechanism underlying Bcl7a-mediated neuronal dysfunction, our *in vivo* findings clearly indicate that the loss of one single SWI/SNF component (i.e., Bcl7a) cannot be compensated for by other alternative chromatin remodeling complexes or variants^12,20,30^. Importantly, our data reveal that Bcl7a contributes to cerebellar plasticity, whereas neuron-specific loss of Bcl7a and Bcl7b has no major effect on spatial memory-related tasks, expanding our knowledge about the specialized roles of SWI/SNF assemblies in high-order brain functions.

## Experimental procedures

### Animal work

All experimental procedures were performed in accordance with the institutional animal welfare guidelines and were approved by the State Agency for Nature, Environment and Consumer Protection of North Rhine-Westphalia, Germany. Mice were kept in groups of three in individual ventilated cages (Tecniplast) under standard ambient conditions (22 °C, 60% humidity) and a 12-hour light/dark-cycle (lights on at 6 a.m.). They had *ad libitum* access to rodent chow (ssniff**®** V1534-300) and tab water. All behavioral testing was done during the animals’ light phase between 9:00 and 17:00.

### Antibodies

The following primary antibodies were used: anti-Bcl7a (mouse monoclonal mAb: 15 C/H4; WB 1:100, IHC 1:20); anti-Bcl7a (rabbit, Atlas antibodies; WB 1:1000, IHC 1:250); anti-Bcl7b (rabbit, Proteintech; WB 1:1000, IHC 1:250); anti-Calbindin (rabbit, Swant; 1:1000); anti-Map2 (rabbit, Millipore; IHC 1:500); anti-tubulin (SIGMA; WB 1:10000), anti-βIII-tubulin (Promega; IHC 1:500). Goat anti-rabbit and goat anti-mouse HRP-conjugated secondary antibodies were from Promega (Madison, WI, USA; WB 1:2000) and Pierce (Darmstadt, Germany; WB 1:2000) respectively. Goat anti-mouse and anti-rabbit Alexa Fluor-conjugated secondary antibodies were from Life Technologies (Darmstadt, Germany; IHC 1:500).

### Behavioral tests

Behavioral analysis of *Bcl7b* ^*KO/KO*^ animals was done in male and female mice. *Bcl7b* ^*wt/wt*^, CMV-Cre^wt/wt^ and *Bcl7b* ^*wt/wt*^, CMV-Cre^tg/wt^ animals were used as littermate controls. In the case of conditional *Bcl7a* ^*nKO*^ and *Bcl7* ^*nDKO*^ mice, behavioral tests were performed with males, and littermates carrying the floxed alleles but not the Synapsin-Cre transgene were used as controls. Prior to behavioral testing, mice were handled daily for seven days. Testing started when the mice were between 10 and 12 weeks old. To minimize the effects of previous tests on subsequent behavior, we performed the behavioral test battery in a specific order in which the less stressful tests preceded the more stressful ones. The individual behavioral tests were separated by one week. *Open field*. Spontaneous locomotor activity and exploratory behavior was assessed in open field boxes (27 cm × 27 cm × 27 cm) in an evenly lit room. Each mouse was placed individually in the centre of the box and its behavior was recorded for 20 min. The distance moved (cm), velocity (cm/s) as well as the time (s) spent in the centre of the box (a commonly used index for anxiety) were analysed using the EthoVision tracking system (Noldus, The Netherlands). *Catwalk*. Gait analysis of freely moving mice was performed with the CatWalk system (Noldus). Briefly, the CatWalk instrument consists of a glass walkway enclosed by dark plastic walls, a fluorescence lamp which emits light inside the glass plate, a high speed colour camera as well as a recording and analysis software. The recordings were carried out in the dark. Each mouse was placed individually in the CatWalk walkway and allowed to move freely and traverse from one side to the other. Where the mouse paw made contact with the glass plate, light was reflected down and the illuminated contact areas were recorded with a high-speed camera positioned underneath the glass plate. Mice were tested over four consecutive days with three blocks of two runs per day. After each block of runs, the animals were put back into their home cages and allowed to rest for 90 min. Trials in which the mouse stopped or changed the direction were excluded from subsequent analysis, and six uninterrupted trials with a maximum speed variation of 30% were analysed and averaged to obtain the final gait analysis value. Parameters such as stride length (cm), swing speed (cm/s), stand and paw overlap were measured using the CatWalk system software (Noldus). *Rotarod*. Motor coordination was assessed using a rotarod system (TSE Systems, Bad Homburg, Germany). Mice were trained to remain on the rod, which rotated with accelerating speed (4 to 40 rpm), over four consecutive days with three daily sessions and a maximum session duration of 5 min. A 30 min break was given between individual training sessions. When a mouse fell from the rod, infrared light beams at the bottom of the chamber were interrupted allowing for the measurement of the fall latency (s). *Morris water maze*. MWM is a commonly used behavioral test to assess hippocampus-dependent spatial learning and memory in rodents. Here, the test apparatus consisted of a white circular swimming pool (150 cm in diameter) filled with water that was maintained at 22 °C and made opaque with white paint. A transparent Plexiglas platform was positioned in a fixed location in the pool and submerged 0.5 cm below the water surface. Mice were trained to locate the platform position over five consecutive days with two to three blocks of two trials per day and a maximum trial duration of 60 s. Between each block of trials, animals were allowed to rest for at least 90 min. The starting positions were semi-randomized so that each trial started from a different quadrant. Short-term memory was tested in a 60 s probe trial 90 min after the last block of training trials on day 3, while long-term memory was assessed on day 6, 24 h after the last training session. During training sessions, the latency to find the platform (escape latency, s), the swim speed (cm/s) as well as the swim distance (cm) were analyzed using a video tracking system (EthoVision, Noldus, The Netherlands). Memory performance during probe trials was assessed via the time spent in the target compared to non-target quadrants (quadrant occupancy) as well as the number of platform position crossings. *Three-chambered social interaction test*. The three-chambered social test apparatus consisted of an acrylic box (63 cm × 42 cm × 23 cm) that was divided into three chambers with two acrylic walls. The dividing walls had retractable doors allowing access into each chamber. A wire cup made out of cylindric chrome bars (10 cm H, 10 cm in diameter) was used to restrain the stranger mouse. At the beginning of each trial, test mice were confined in the centre chamber, and a trial was initiated by opening the doors to the side chambers. During the habituation phase, an empty wire cup was placed into each side chamber and the mouse was allowed to explore all three chambers freely for 10 min. Thereafter, the mouse was placed back into the centre chamber with the doors to the side chambers closed. For the subsequent social interaction trial, an unfamiliar mouse was placed into the wire cup in one of the side chambers while an object was placed into the cup of the other side chamber. The location of the social interaction partner and the object were pseudo-randomized throughout the test sessions. The social interaction trial lasted 10 min, and the time spent in each chamber was video-recorded and analysed by an automatic tracking program (EthoVision, Noldus, The Netherlands). All stranger mice were habituated to being enclosed in the wire cups for 5 min daily two days before the experiment. *Contextual fear conditioning*. Fear conditioning was conducted in transparent plastic boxes (21.5 × 20 × 25 cm) with stainless-steel grid floors connected to an aversive stimulator (Med Associates). For fear conditioning, mice were placed individually into the chamber and allowed to habituate for 2 min. Thereafter, three shocks (0.75 mA, 2 s) with an inter-shock interval of 60 s were delivered. Animals were removed from the boxes 60 s after the last shock and placed back into their home cages. In order to examine memory retrieval, mice were re-introduced into the chambers 24 h after the conditioning procedure for 5 min without the presentation of foot shocks. During fear conditioning and retrieval, mice were video recorded and the time spent freezing was analysed.

### Cell culture

Primary cortical neurons were isolated from E16.5/17.5 embryos and maintained in Neurobasal medium (Invitrogen) supplemented with 2% glutamax and 2% B27 supplement (Invitrogen) as previously described^49,50^.

### Golgi Staining

Animals were sacrificed via cervical dislocation; brains were rapidly removed from the scull and stained with the FD rapid Golgistain_TM_ Kit (FD NeuroTechnologies) according to the manufacturer’s instructions. Sagittal brain sections were cut on a cryostat at a thickness of 100 μm. Bright-field images were taken using a Zeiss EPI-SCOPE1 Apotome equipped with a 20x objective. Sholl analysis was done in ImageJ, and branch intersections were quantified at 10μm-spaced concentric circles starting from the cell soma.

### Generation of Bcl7a^fl/fl^ and Bcl7b^fl/fl^ transgenic mice

*Bcl7a* ^*fl/fl*^ and *Bcl7b* ^*fl/fl*^ mice were generated by the inGenious Targeting Laboratory (USA). For both lines, we designed a gene targeting strategy that flanked exon 2 of the *Bcl7a* and *Bcl7b* gene with loxP sites, which upon Cre recombination resulted in the deletion of exon 2 and a premature stop codon in exon 3. The use of this targeting strategy enabled the ablation of all transcripts known to encode for functional proteins of *Bcl7a* and *Bcl7b*, respectively. For the generation of floxed *Bcl7a* and *Bcl7b* mice, targeting vectors were transfected via electroporation in C57BL/6 embryonic stem cells and positive clones containing the targeted alleles were confirmed via Southern Blot. Targeted embryonic stem cells were then microinjected into Balb/c blastocysts and subsequently transmitted into the germ line. Homozygous *Bcl7a* ^*fl/fl*^ and *Bcl7b* ^*fl/fl*^ mice were generated through appropriate crosses and kept on a C57BL/6 background. For the generation of *Bcl7a* and *Bcl7b* full knockout mice, *Bcl7a* ^*fl/fl*^ and *Bcl7b* ^*fl/fl*^ males were first crossed with females carrying the CMV-Cre transgene, resulting in the ubiquitous deletion of the loxP site flanked genes^51^. Subsequent crosses of heterozygous animals were then performed to generate *Bcl7a* and *Bcl7b* full knockout mice. Tissue samples from *Bcl7a* ^*KO/KO*^ and *Bcl7b* ^*KO/KO*^ embryos were used for Sanger sequencing which confirmed the deletion of exon 2 of the *Bcl7a* and *Bcl7b* gene, respectively (data not shown). Conditional *Bcl7a* and *Bcl7a;Bcl7b* knockout animals were generated via crosses of *Bcl7a* ^*fl/fl*^ and *Bcl7a* ^*fl/fl*^ *;Bc7b* ^*fl/fl*^ males with females carrying the Synapsin-Cre transgene which causes the neuronal-specific deletion of loxP site flanked genes^36^. CMV- and Synapsin-Cre deleter lines were purchased from the Jackson Laboratory (USA, JAX stock numbers: JAX006054, JAX003966). All mouse lines were kept on the C57BL/6 background.

### Immunostaining and confocal analysis

Free-floating brain sections were incubated in blocking solution (10% NGS, 0.5% Triton-X 100 in PBS) for 1 hour at room temperature. Incubation with primary antibodies was done in blocking solution for 48–72 hours at 4 °C. Sections were incubated with secondary antibodies after 3 washes in PBS for 2 hours at room temperature or for 48 hours at 4 °C. After 3 steps of washing in PBS, slices were stained with Hoechst (1:10000), mounted and air dried overnight. Slices were finally stained with Sudan black (0.3% in 70% ethanol) to reduce autofluorescence, and mounted with Dako Fluorescent Mounting Media (S3023, Agilent). Images were acquired on a laser scanning confocal microscope (LSM 700; Carl Zeiss Inc., Germany). For diaminobenzidine (DAB) staining, biotinylated secondary antibodies were used. Following incubation with Vector Elite ABC kit, sections were developed with 3-3′ diaminobenzidine (DAB), dehydrated and mounted with DePex Mounting Media (VWR). Bright-field images were taken with a Zeiss EPI-SCOPE1 Apotome equipped with a 20x objective. Counting of cabindin-positive cells was performed on three consecutive sections per animal by an investigator blinded to genotype groups.

### RNA extraction and absolute quantification by qRT-PCR

RNA from mouse tissues was extracted using a RNA extraction kit (QIAGEN) and following the manufacturer instructions. Isolated mRNA was retro-transcribed with qScript cDNA SuperMix (Quanta Biosciences). All RT-PCR experiments were performed using fast SYBR green master mix (Applied Biosystem) and reaction was carried out in a StepOne plus Thermocycler (Applied Biosystem). Primers used for qRT-PCR (ΔΔCt) for mouse were: Bcl7a F gagaagaaatgggtgaccgt and R tcgtccttgcctttcttcttg; Bcl7b F gggagaagaaatgggtgactg and R tccttgctatctgtcacaggc; Bcl7c F gggccaaagatgacatcaag and R gcacccacttgaagattcga. Absolute quantification by qRT-PCR was performed using 60 ng of cDNA as a template for conventional PCR. The following primers were used: Bcl7a F tgatatcaagagggtcatggc and R ttgctgttatcatcgtgcatgt; Bcl7b F gtagccgggctaaagatgacat and R tctggttgctgttctcatcctg; Bcl7c F atggccggccggaccgt and R tctgccccgcctcctgcc. PCR product was then purified, quantified and the copy number/μl calculated. The product was then diluted to approx. 10,000 copies/μl, 1:2 serial dilutions made and qRT-PCR was performed with Fast SYBR Green Master Mix on a Step One Plus Real Time PCR System in order to generate a standard curve. qRT-PCR was then performed on 5 ng of cDNA retrotranscribed from wt mouse tissue samples with primers for comparative RT-PCR, and Ct values were compared to the standard curve to estimate copy number/μl of genes of interest.

### Tissue processing

For RNA extraction, mice were sacrificed via cervical dislocation. Brains were rapidly dissected, snap frozen in liquid nitrogen and stored at −80 °C until further processing. For histology and immunohistochemistry, mice were anesthetized with an overdose of ketamine/xylazine and transcardially perfused with phosphate-buffered saline (PBS) followed by 4% paraformaldehyde. Brains were removed from the scull and transferred into a 30% sucrose solution for 72 h. Six series of coronal brain sections (40 μm) were cut on a cryostat (Thermo Scientific) and immunohistochemistry was performed on free-floating sections.

### Statistical analysis

Data are expressed as mean ± S.E.M. and statistical analysis were performed with GraphPad Prism Software (GraphPad Software Inc., San Diego, USA). Behavioral data were analysed using two-tailed, unpaired Student’s t-tests or two-way analysis of variance (ANOVA) with repeated measures (RM) and post hoc Bonferroni multiple comparisons. Sholl analysis data were analysed via two-way RM ANOVA with genotype as between-subjects factor and radius as the within-subject repeated measures factor. The statistical significance was defined as *p* < 0.05.

### Western Blotting

Proteins were transferred on nitrocellullose membranes using a semidry transfer system (Biorad). Filters were blocked for 1 hour at room temperature in 5% non-fat dry milk (Applichem) in TBST (Tris buffered saline +0.05% Tween-20). Primary antibodies were incubated overnight at 4 °C. Membranes were washed in TBST for 15 min and then incubated with the matching polyclonal HRP-conjugated antibodies for 1 hour at room temperature. Blots were exposed with ECL substrate and chemiluminescent signals detected using a CCD camera-equipped ChemiDoc XRS system (Biorad). Densitometry was performed using the Chemidoc software Imagelab.

## Electronic supplementary material

Supplementary Information
