# Supplementary material for: The SWI/SNF subunit Bcl7a contributes to motor coordination and Purkinje cell function
Source: Sci Rep. 2017 Dec 6;7:17055. doi: 10.1038/s41598-017-17284-3 (PMC5719005; doi:10.1038/s41598-017-17284-3)
Supplement: Supplementary file 1 — Supplementary Information [file 41598_2017_17284_MOESM1_ESM.pdf]

Supplementary Information

Title: The SWI/SNF subunit Bcl7a contributes to motor coordination and Purkinje cell function.

Author list: Lena Wischhof, Simona Maida, Antonia Piazzesi, Anna Gioran, Kristina Barragan Sanz, Stephan Irsen, Marc Beyer, Joachim L. Schultze, Martin J. Dyer, Paolo Salomoni, Dan Ehninger, Pierluigi Nicotera, and Daniele Bano

A

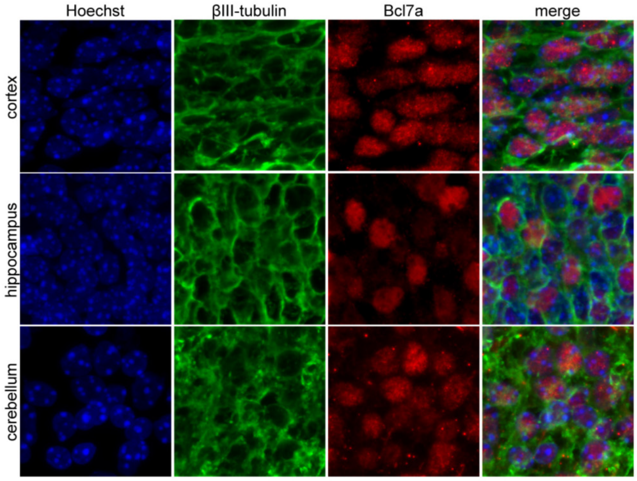

B

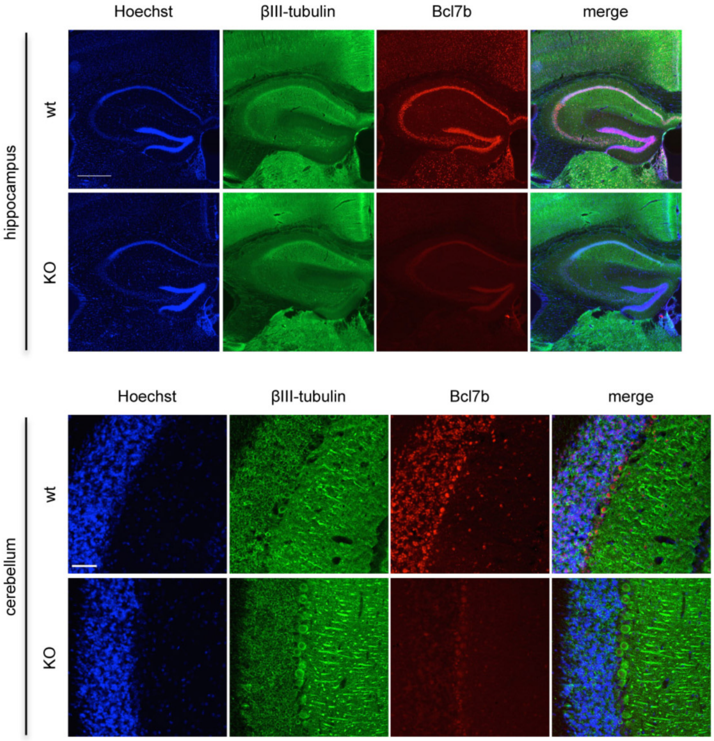

C

	**Embryos**	**Adults**
Bcl7a$^{wt/wt}$	32.4%	28.8%
Bcl7a$^{KO/wt}$	41.9%	67.2%
Bcl7a$^{KO/KO}$	25.7%	4.0%
**N. animals**	74	248

Supplemental Figure S1 (relative to Figure 1). (A) Immunofluorescence images of Bcl7a in the early postnatal mouse brain. Panels show Bcl7a expression throughout various brain regions and the nuclear localization of Bcl7a (red). Hoechst-33342 (blue) and β-III tubulin (green) were used as nuclear and neuronal markers, respectively. (B) Bcl7b immunostaining in hippocampi (upper panel) and cerebella (lower panels) from wild type and $Bcl7b^{KO/KO}$ adult mice (blue: Hoechst-33342; green: β-III tubulin; red: Bcl7b). Scale bars=500 µm for upper and 50 µm for lower panels. (C) $Bcl7a^{KO/KO}$ embryos were found at the expected Mendelian ratio, whereas only a few animals survive into adulthood.

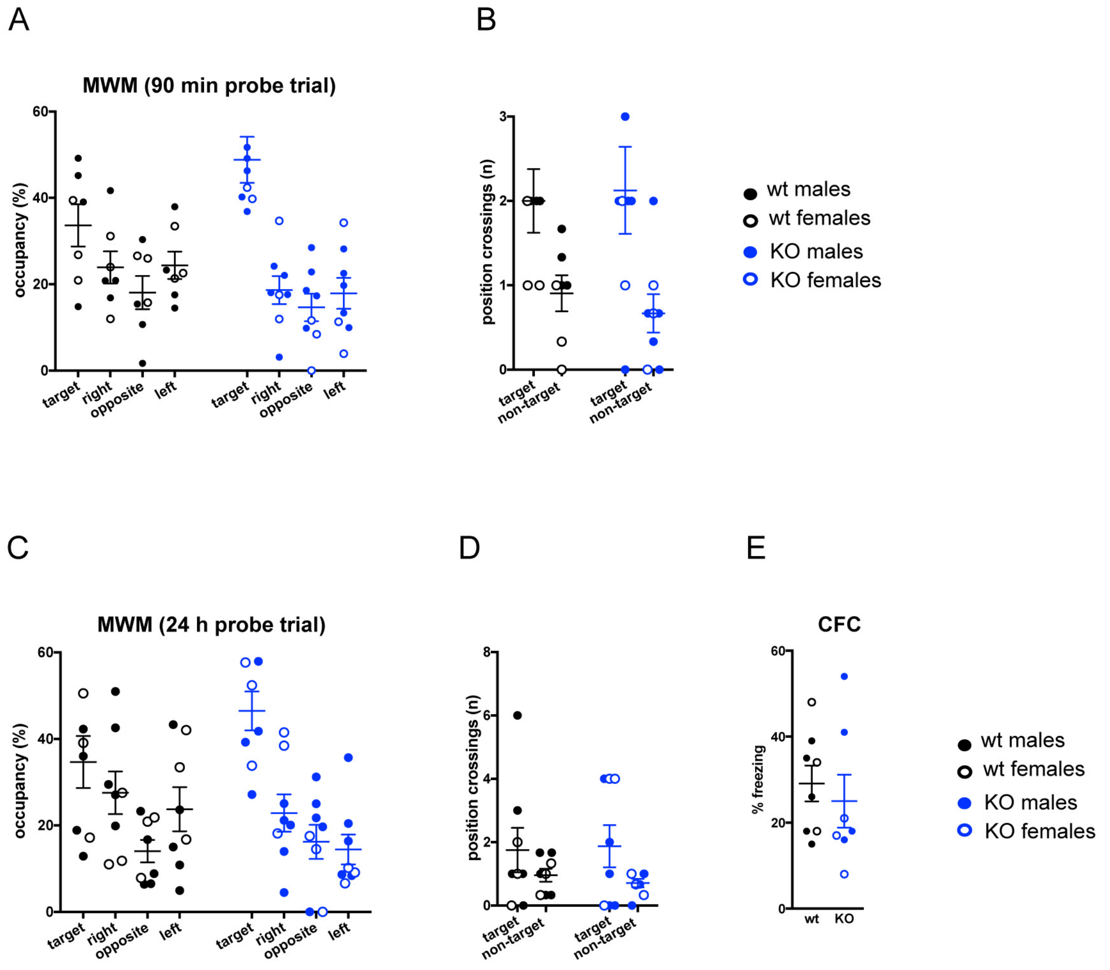
A
B
MWM (90 min probe trial)
occupancy (%)
60
40
20
0
target
right
opposite
left
target
right
opposite
left
position crossings (n)
3
2
1
0
target
non-target
target
non-target
wt males
wt females
KO males
KO females
C
D
E
MWM (24 h probe trial)
occupancy (%)
60
40
20
0
target
right
opposite
left
target
right
opposite
left
position crossings (n)
8
6
4
2
0
target
non-target
target
non-target
CFC
% freezing
60
40
20
0
wt
KO
wt males
wt females
KO males
KO females

Supplementary Figure S2 (relative to Figure 2). (A-B) In the Morris water maze (MWM) probe trial, performed 90 min after the last training session on day 3, no differences were seen between male and female wild type (wt; n=7 (4 ♂, 3 ♀)) and *Bcl7b*$^{KO/KO}$ (KO; n=8 (5 ♂, 3 ♀)) mice regarding the time spent in the target quadrant (A). Compared to wt males, wt females showed a slight reduction the number of target position crossings which was not seen in the KO group (B). (C- D) Both wt (n=8 (5 ♂, 3 ♀)) and KO (n=8 (5 ♂, 3 ♀)) male and female mice did not perform significantly different in terms of target quadrant occupancy (C) and target position crossings (D) in the long-term memory probe trial, performed 24 h after the last training trial on day 4. (E) In the contextual fear conditioning (CFC) paradigm, no significant sex differences were seen during fear memory retrieval assessed 24 h after the conditioning trial in wt (n=8 (5 ♂, 3 ♀)) and KO (n=8 (5 ♂, 3 ♀)) male and female mice.

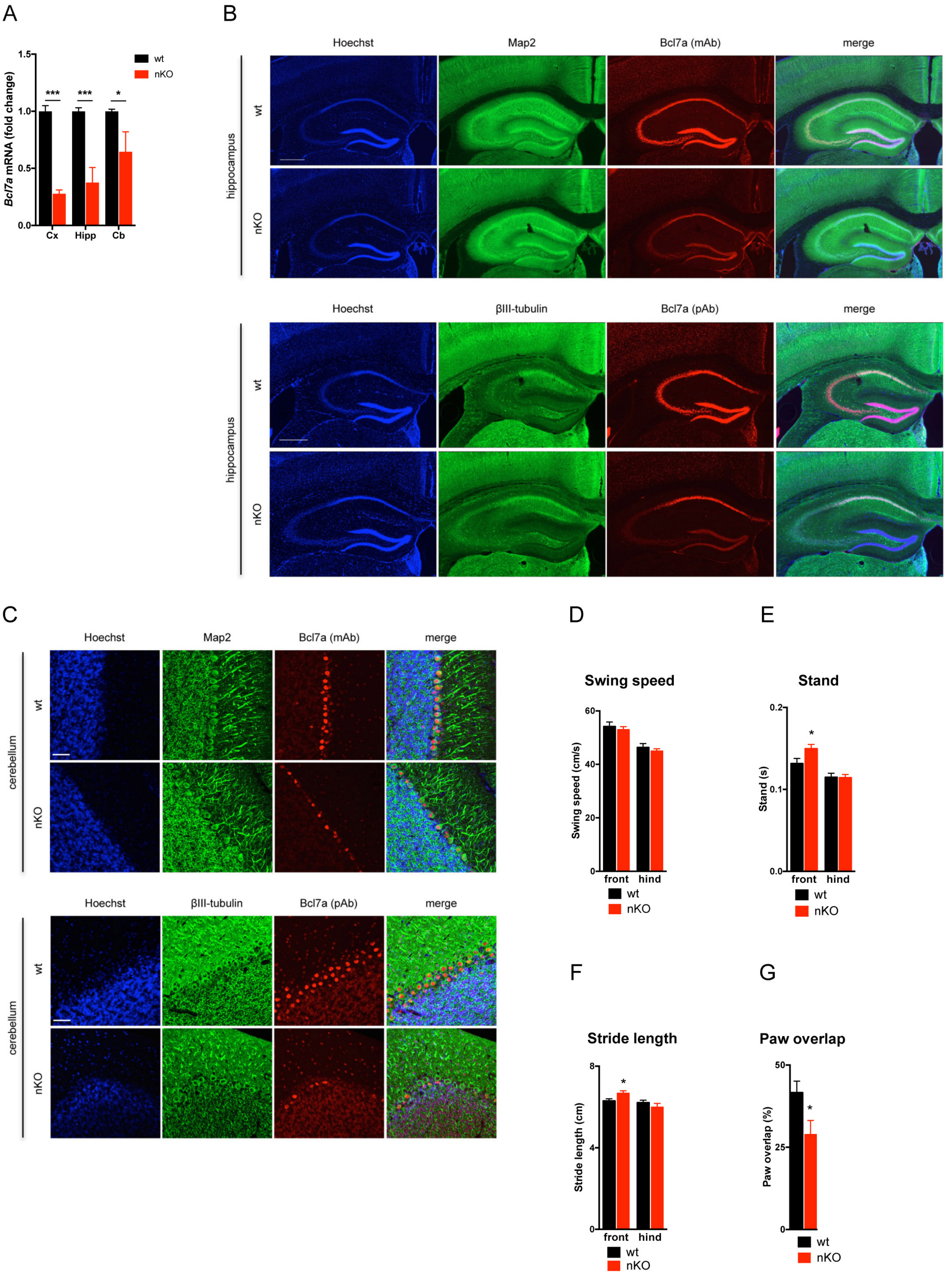

A
B
C
D
E
F
G
Bcl7a mRNA (fold change)
wt
nKO
Cx
Hipp
Cb
Hoechst
Map2
Bcl7a (mAb)
merge
βIII-tubulin
Bcl7a (pAb)
hippocampus
cerebellum
Swing speed
Swing speed (cm/s)
Stand
Stand (s)
Stride length
Stride length (cm)
Paw overlap
Paw overlap (%)
front
hind

Supplemental Figure S3 (relative to Figure 3). (A) qRT-PCR of *Bcl7a* mRNA levels in adult brains from wild type and $Bcl7a^{nKO}$ animals (mean ± S.E.M.; *p<0.05, ***p<0.0001). *Bcl7a* mRNA levels were significantly reduced throughout the cortex (Cx), hippocampus (Hipp) and cerebellum (Cer) of $Bcl7a^{nKO}$ animals. (B-C) Immunohistochemical analyses of Bcl7a in (B) hippocampi and (C) cerebella from $Bcl7a^{nKO}$ and control mice (wt) using Bcl7a-specific mono- and polyclonal antibodies. βIII-tubulin and MAP2 were used as neuronal marker (blue: Hoechst-33342; green: β-III tubulin; red: Bcl7a). Bcl7a monoclonal (mAb: 15C/H4) and polyclonal (pAb: Atlas antibodies) antibodies (mAb) were used as indicated. Scale bar = (B) 500 μm and (C) 50 μm. (D-G) Catwalk gait analysis in $Bcl7a^{nKO}$ mice. (D) While the swing speed of front and hind paws did not differ between wild type (n=14) and $Bcl7a^{nKO}$ (n=14) animals, a significant increase was seen in front paw stand duration (E, *t* test p=0.0349) and stride length (F, *t* test p=0.0199). (G) Further gait abnormalities in $Bcl7a^{nKO}$ animals were detected by the reduced paw print overlap (*t* test p=0.029).

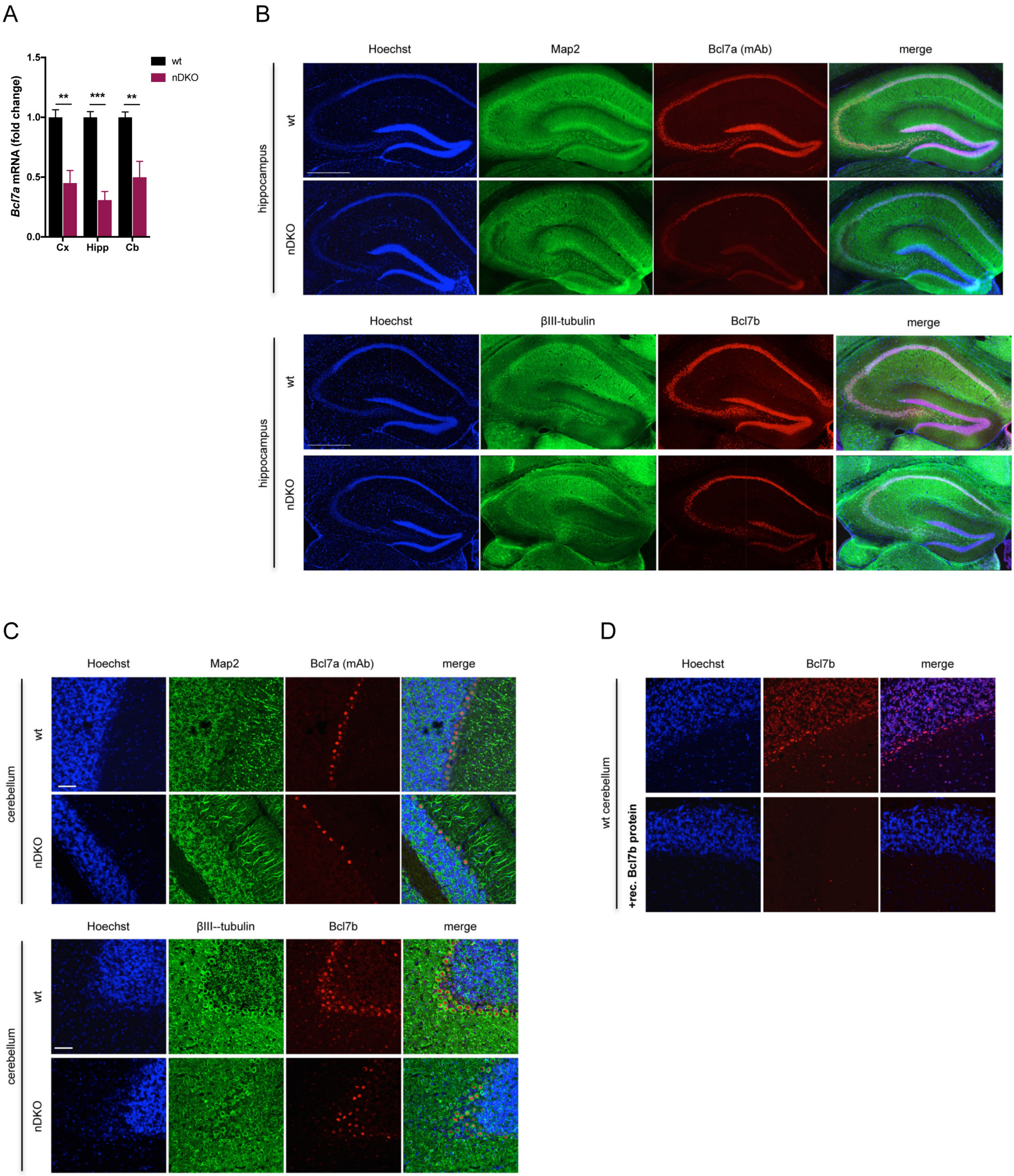

A
B
C
D
Bcl7a mRNA (fold change)
1.5
1.0
0.5
0.0
wt
nDKO
**
***
**
Cx
Hipp
Cb
Hoechst
Map2
Bcl7a (mAb)
merge
hippocampus
wt
nDKO
Hoechst
βIII-tubulin
Bcl7b
merge
hippocampus
wt
nDKO
Hoechst
Map2
Bcl7a (mAb)
merge
cerebellum
wt
nDKO
Hoechst
βIII--tubulin
Bcl7b
merge
cerebellum
wt
nDKO
Hoechst
Bcl7b
merge
wt cerebellum
+rec. Bcl7b protein

Supplemental Figure S4 (relative to Figure 4). (A) qRT-PCR of *Bcl7a* mRNA levels in cortex (Cx), hippocampus (Hipp) and cerebellum (Cb) from wild type and $Bcl7^{nDKO}$ animals (mean ± S.E.M.; $^{**}p<0.001$, $^{***}p<0.0001$). (B-D) Immunohistochemical analyses of Bcl7a and Bcl7b in $Bcl7^{nDKO}$ and control mice (wt). bIII-tubulin and MAP2 were used as neuronal marker. (B) Images show the loss of Bcl7a and Bcl7b positive cells within neuronal populations of the hippocampus in $Bcl7^{nDKO}$ animals. Scale bar=500 µm. (C) Likewise, Bcl7a and Bcl7b immunoreactivity was reduced within most neurons of the cerebellum in $Bcl7^{nDKO}$ animals. Scale bars = 50 µm. (D) Immunohistochemical analyses with Bcl7b antibody in cerebella slices (blue: Hoechst-33342; red: Bcl7b). Pre-absorption with recombinant Bcl7b protein abolished immunoreactivity of Bcl7b antibody.

Supplemental Figure S5: uncropped immunoblots, relative to Figure 1I

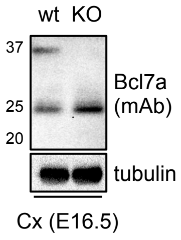

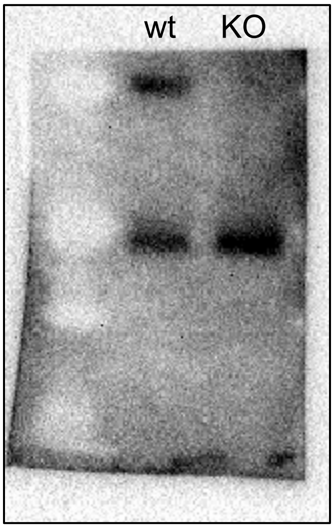

Bcl7a
(mAb)

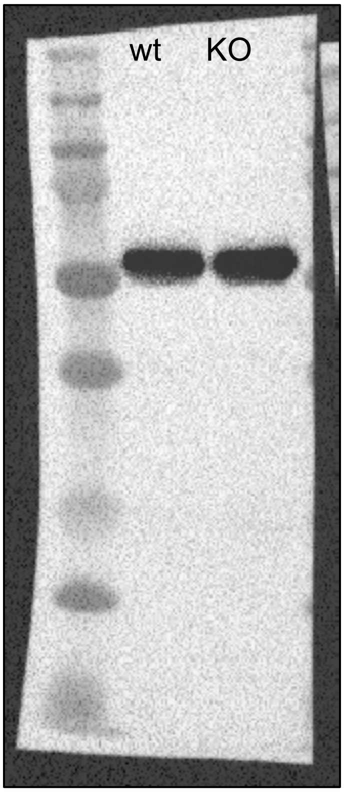

tubulin

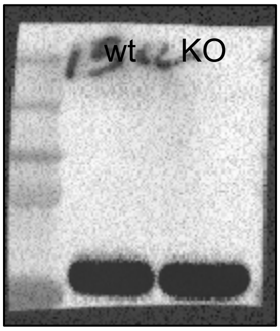

tubulin

Supplemental Figure S6: uncropped immunoblots, relative to Figure 1J

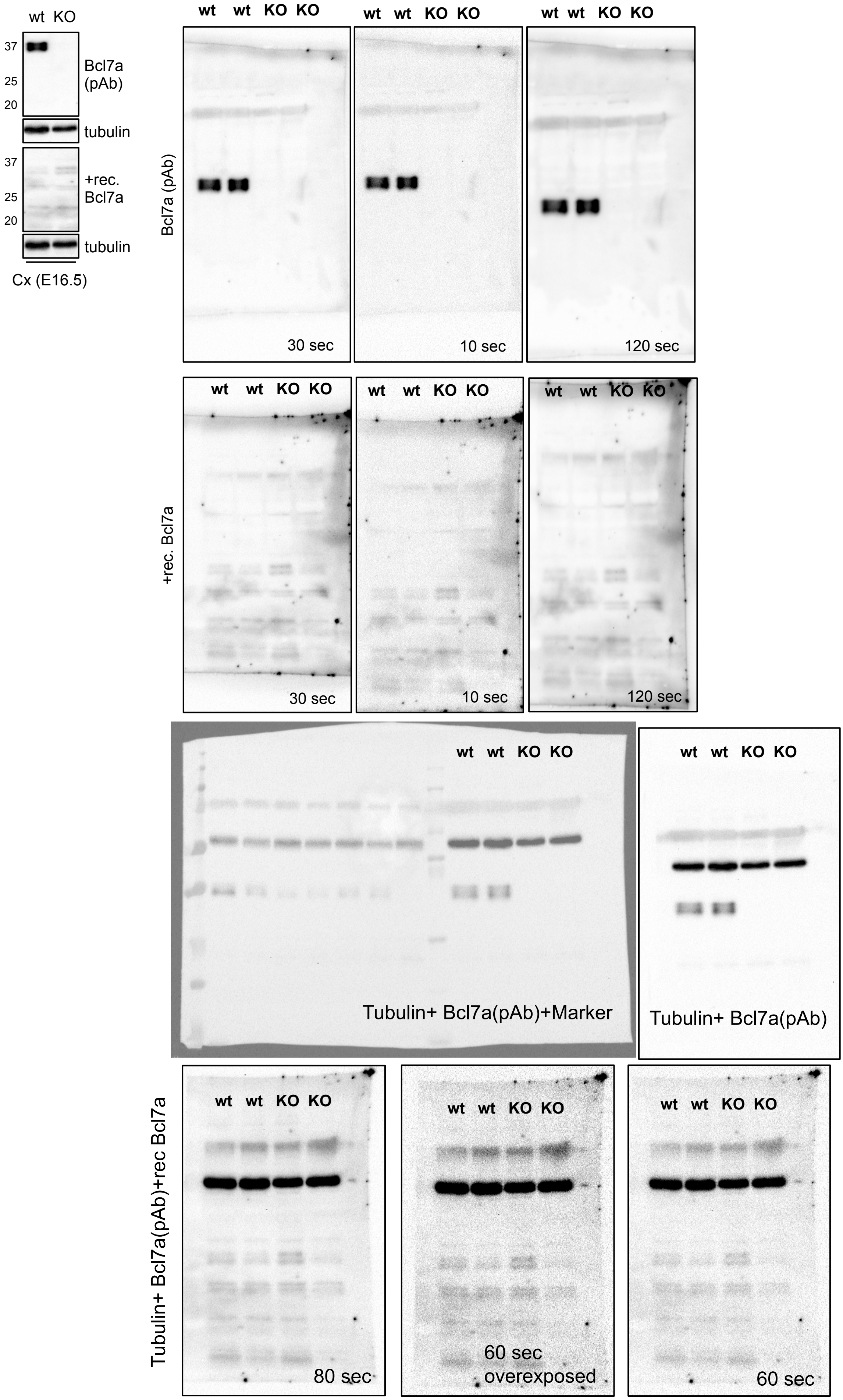

Supplemental Figure S7: uncropped immunoblots, relative to Figure 1K

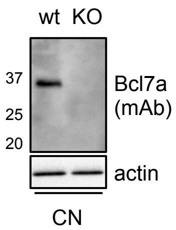

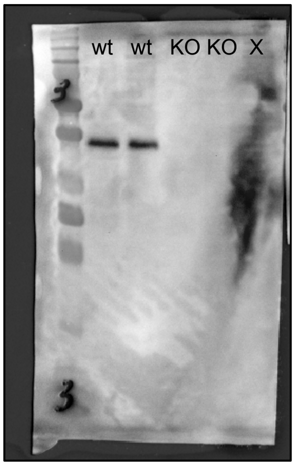

Bcl7a
(mAb)

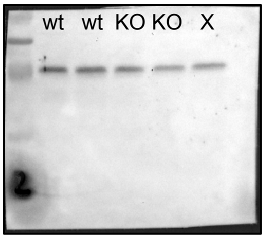

actin

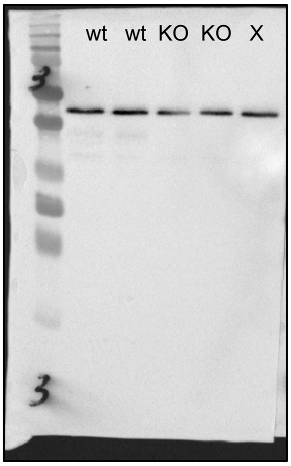

actin
[on filter previously
exposed to Bcl7a (mAb)]

Supplementary Table 1. Statistical analysis of sex differences in *Bcl7b*$^{wt/wt}$ and *Bcl7b*$^{KO/KO}$ mice as well as of potential effects of the CMV-Cre transgene in the control group.

Measure	Genotype	Test	Factor	F (DFn, Dfd)	*p*-value
**Open field**					
distance moved	wt	Two-way RM ANOVA	Sex<br>Time interval<br>Interaction	F (1, 6)=3.975<br>F (19,114)=2.951<br>F (19,114)=1.364	0.093<br><0.001<br>0.115
distance moved	KO	Two-way RM ANOVA	Sex<br>Time interval<br>Interaction	F (1, 6)=0.035<br>F (19,114)=2.05<br>F (19,114)=0.908	0.856<br>0.002<br>0.605
average velocity	wt	*t*-test	males vs. females		0.092
average velocity	KO	*t*-test	males vs. females		0.844
distance moved	wt	Two-way RM ANOVA	Transgene<br>Time interval<br>Interaction	F (1, 6)=2.413<br>F (19,114)=1.36<br>F (19,114)=0.732	0.171<br>0.161<br>0.778
average velocity	wt	t-test	*CMV-Cre*$^{wt/wt}$ vs. *CMV-Cre*$^{tg/wt}$		0.193
**RotaRod**					
fall latency	wt	Two-way RM ANOVA	Sex<br>Day<br>Interaction	F (1, 6)=0.121<br>F (3, 18)=0.025<br>F (3, 18)=0.967	0.739<br>0.994<br>0.429
fall latency	KO	Two-way RM ANOVA	Sex<br>Day<br>Interaction	F (1, 6)=0.447<br>F (3, 18)=2.007<br>F (3, 18)=0.178	0.528<br>0.149<br>0.909
fall latency	wt	Two-way RM ANOVA	Transgene<br>Day<br>Interaction	F (1, 6)=0.024<br>F (3, 18)=0.272<br>F (3, 18)=0.524	0.879<br>0.844<br>0.671
**MWM (90min)**					
Quadrant occupancy (90min delay)	wt	Two-way RM ANOVA	Sex<br>Quadrant<br>Interaction	F (1, 5)=0.304<br>F (3, 15)=1.543<br>F (3, 15)=0.524	0.605<br>0.244<br>0.672
Quadrant occupancy (90min delay)	KO	Two-way RM ANOVA	Sex<br>Quadrant<br>Interaction	F (1, 6)=1.183<br>F (3, 18)=16.78<br>F (3, 18)=2.531	0.318<br>0.001<br>0.089
Target crossings	wt	Two-way RM	**Sex**<br>Position	**F (1, 5)=9.6**<br>F (1, 5)=7.771	**0.026**<br>0.038

(90min delay)		ANOVA	Interaction	F (1, 5)=0.221	0.657
Target<br>crossings<br>(90min delay)	KO	Two-way<br>RM<br>ANOVA	Sex<br>Position<br>Interaction	F (1, 6)=0.255<br>F (1, 6)=9.695<br>F (1, 6)=1.047	0.644<br>0.190<br>0.826
Quadrant<br>occupancy<br>(90min delay)	wt	Two-way<br>ANOVA	Transgene<br>Quadrant<br>Interaction	F (1, 5)=0.240<br>F (3, 15)=1.802<br>F (3, 15)=0.298	0.631<br>0.020<br>0.345
Target<br>crossings<br>(90min delay)	wt	Two-way<br>ANOVA	Transgene<br>Position<br>Interaction	F (1, 5)=0.095<br>F (1, 5)=10.56<br>F (1, 5)=0.300	0.770<br>0.022<br>0.607

**MWM<br>(24h)**					
Quadrant<br>occupancy<br>(24h delay)	wt	Two-way<br>RM<br>ANOVA	Sex<br>Quadrant<br>Interaction	F (1, 6)=2.728<br>F (3, 18)=2.104<br>F (3, 18)=1.139	0.149<br>0.135<br>0.360
Quadrant<br>occupancy<br>(24h delay)	KO	Two-way<br>RM<br>ANOVA	Sex<br>Quadrant<br>Interaction	F (1, 6)=3.725<br>F (3, 18)=10.86<br>F (3, 18)=1.598	0.101<br><0.001<br>0.224
Target<br>crossings<br>(24h delay)	wt	Two-way<br>RM<br>ANOVA	Sex<br>Position<br>Interaction	F (1, 6)=1.227<br>F (1, 6)=0.503<br>F (1, 6)=0.346	0.310<br>0.504<br>0.577
Target<br>crossings<br>(24h delay)	KO	Two-way<br>RM<br>ANOVA	Sex<br>Position<br>Interaction	F (1, 6)=0.813<br>F (1, 6)=3.103<br>F (1, 6)=0.775	0.401<br>0.128<br>0.412
Quadrant<br>occupancy<br>(24h delay)	wt	Two-way<br>ANOVA	Transgene<br>Quadrant<br>Interaction	F (1, 6)=1.476<br>F (3, 18)=2.042<br>F (3, 18)=0.232	0.270<br>0.143<br>0.872
Target<br>crossings<br>(24h delay)	wt	Two-way<br>ANOVA	Transgene<br>Position<br>Interaction	F (1, 6)=5.941<br>F (1, 6)=2.291<br>F (1, 6)=0.3.483	0.050<br>0.180<br>0.111

**FC**					
Freezing<br>(baseline)	wt<br>KO	Two-way<br>ANOVA	Sex<br>Genotype<br>Interaction	F (1, 12)=2.749<br>F (1, 12)=0.006<br>F (1, 12)=1.067	0.123<br>0.938<br>0.322
Freezing<br>(retrieval)	wt<br>KO	Two-way<br>ANOVA	Sex<br>Genotype<br>Interaction	F (1, 12)=0.146<br>F (1, 12)=1.775<br>F (1, 12)=1.516	0.708<br>0.207<br>0.241
Freezing<br>(baseline)	wt	*t*-test	*CMV-Cre*$^{wt/wt}$ vs.<br>*CMV-Cre*$^{tg/wt}$		0.276
Freezing<br>(retrieval)	wt	*t*-test	*CMV-Cre*$^{wt/wt}$ vs.<br>*CMV-Cre*$^{tg/wt}$		0.476
